# The promoter -1031(T/C) polymorphism in *tumor necrosis factor-alpha *associated with polycystic ovary syndrome

**DOI:** 10.1186/1477-7827-9-131

**Published:** 2011-10-04

**Authors:** Ji-Hyun Yun, Jin-Woo Choi, Kyung-Ju Lee, Joong-Sik Shin, Kwang-Hyun Baek

**Affiliations:** 1 Department of Biomedical Science, CHA University, Bundang CHA General Hospital, Gyeonggi-Do, Republic of Korea; 2 St. Paul's School, Concord, NH, USA; 3 Department of Obstetrics and Gynecology, CHA General Hospital, Seoul, Korea

**Keywords:** Polycystic ovary syndrome, TNF-alpha, tumor necrosis factor, PCR-RFLP

## Abstract

**Background:**

A *tumor necrosis factor-alpha *is a multifunctional pro-inflammation cytokine, which has been considered as one of pathogenic factors for various diseases. The promoter -1031(T/C) polymorphism in the *tumor necrosis factor-alpha *gene was reported that it plays a part in reproduction-related diseases. Among these, polycystic ovary syndrome (PCOS) is known to be a common gynecological disease of women in reproductive age women. Here, we performed a comparative study of -1031(T/C) polymorphism of *TNF-alpha *gene with PCOS in a Korean population.

**Methods:**

The -1031(T/C) polymorphism of *TNF-alpha *gene was analyzed by polymerase chain reaction restriction fragment length polymorphism (PCR-RFLP) in a total of 217 PCOS patients and 144 matched female controls of healthy women. And statistical analysis was performed using HapAnalyzer. *X*^2 ^test and logistic regression were utilized analyze the association between two groups. A *p*-value under 0.05 was considered statistically significant.

**Results:**

The genotype and allelic frequencies were in Hardy-Weinberg equilibrium (HWE). There was strong association between the -1031(T/C) polymorphism in the promoter region of *TNF-alpha *gene and PCOS (*p*-value = 0.0003, odd ratio (OR) = 2.53). In addition, the frequency of C allele was significantly higher in PCOS patients compared with controls. Sequence analyses also showed the -1031(T/C) polymorphism of *TNF-alpha *gene.

**Conclusion:**

This is the first study on the -1031(T/C) polymorphism of *TNF-alpha *gene in PCOS. We concluded that the -1031(T/C) polymorphism of *TNF-alpha *gene is associated with PCOS in a Korean population. Therefore, it is possible that it may be considered as a clinical biomarker to diagnose for PCOS, and is helpful in understanding the etiology for the pathogenesis of PCOS.

## Background

Polycystic ovary syndrome (PCOS) is one the most common gynecological disorder and affects up to 5% women in reproductive ages [1-4]. Generally, PCOS patients show the symptoms of obesity, increased risk of type 2 diabetes mellitus, menstrual irregularity, and anovulation [5-9]. A number of groups focused on the studies for single nucleotide polymorphisms and expected that could be associated with PCOS. However, its etiology is still not fully identified [10,11].

Up to now, several association studies were reported that the some of polymorphisms of tumor necrosis factor-*α *(TNF-*α*) are related with gynecological diseases including pre-eclampsia, endometriosis [12]. It is a multifunctional proinflammation cytokine and has a significant source of genetic variability [13,14]. Many studies suggested that the TNF-*α *may be considered as an immunological and molecular indicator for gynecological-related diseases. A previous study showed that -857(C/T) and -863(C/A) polymorphisms in *TNF-α *gene showed no effect on endometriosis patients in a Japanese population [15]. Additional genetic studies of -238(G/A) and -308(G/A) promoter polymorphisms in a *TNF-α *gene have no association with endometriosis in a Caucasian population [16]. And -308(G/A) promoter polymorphism does not affect genetic susceptibility to polycystic ovaries [17]. In contrast, only -1031(T/C) polymorphism in a TNF-*α *gene plays a part of endometriosis in Asian populations [15,18]. Even though there are no extensive genetic studies performed on PCOS, previous studies suggest that the -1031(T/C) polymorphism in a *TNF-α *gene may affect PCOS. Here, we investigated the relationship between SNP in -1031(T/C) of *TNF-α *gene and PCOS to investigated -1031(T/C) polymorphism in a *TNF-α *gene is associated with PCOS.

## Methods

### Subjects

All samples were recruited from at Fertility Center of CHA General Hospital in Seoul, Korea. The study appraised 217 PCOS patients and 144 healthy Korean women as case and control groups based on the revised diagnostic criteria according to the 2003 ASRM/ESHRE Rotterdam consensus.

Written informed consent was obtained from all of the participating women. Blood samples were collected in tubes containing EDTA as an anti-clotting factor and stored at -20°C until use. Genomic DNA was isolated from the blood samples of PCOS patients and controls. PCOS patients and controls in this study were all Korean women and this study with human blood samples was authorized by an Institutional Review Board (IRB).

### Biochemical determinations

The clinical and biochemical characteristics of the PCOS patients and controls are indicated in Table 1. Blood samples were collected from both PCOS patients and controls. Hormone and glucose levels, including plasma LH, FSH, PRL, E_2_, TSH, testosterone, and DHEA-S were measured as indicators of distinction.

**Table 1:** Clinical and biochemical characteristic of PCOS patients (n = 217) and normal controls (n = 144)

Characteristics	PCOS patients (n = 217)	Controls (n = 144)
Age (y)	32.47 ± 3.04 (29-35)	33.09 ± 3.36 (29-36)
BMI (kg/m2)	34.99 ± 5.84 (24.38-56.23)	18.77 ± 2.13 (15.04-25.08)
Waist/hip ratio (WHR)	1.23 ± 0.09 (1.00-2.24)	0.72 ± 0.06 (0.63-0.82)
FSH (mIU/ml)	8.75 ± 2.75 (1.50-29.98)	5.93 ± 1.64 (2.71-12.22)
LH (mIU/ml)	12.61 ± 9.87 (1.50-37.67)	2.93 ± 1.34 (1.01-6.43)
E_2 _(pg/ml)	64.69 ± 52.11 (7.53-133.36)	29.4 ± 13.7 (3.84-58.05)
Prolactin (ng/ml)	20.22 ± 14.52 (3.46-108.80)	11.08 ± 5.86 (3.71-42.02)
TSH (μIU/ml)	3.39 ± 2.15 (0.69-15.74)	1.7 ± 0.85 (0.02-3.67)
DHEA-S (μg/dl)	298.48 ± 115.34 (68.26-568.41)	142.96 ± 50.22 (60.86-233.11)
Testosterone (ng/ml)	0.87 ± 0.31 (0.09-1.29)	0.22 ± 0.13 (0.01-0.48)

Numerical data were presented as means ± standard deviation (SD). Abbreviations: BMI, Body mass index: WHR, Waist-hip ratio: FSH, Follicle-stimulating hormone: LH, Luteinizing hormone: E2, Estradiol: TSH, Thyroid-stimulating hormone; DHEA-S, Dehydroepiandrosterone-sulfate

### Genetic analysis

To investigate the association between the -1031(T/C) polymorphism of *TNF-a *gene and PCOS, we collected 217 PCOS patients sample and 144 control samples, and polymerase chain reaction restriction fragment length polymorphism (PCR-RFLP) analysis was performed. The -1031(T/C) polymorphism of *TNF-α *gene was amplified by PCR with a set of primers, 5'-TAT GTG ATG GAC TCA CCA GG-3' and 5'-CCT CTA CAT GGC CCT GTC TT-3'. The 30 cycles of PCR was perform at 96°C for 5 min, 94°C for 30 sec, 63°C for 40 sec, and 72°C for 1 min and final cycle of 72°C for 10 min. Amplified PCR products were purified using AccuPrep Bioneer's PCR purification kit (Bioneer, Daejeon, Korea), and digested with *Bbs *I (New England BioLabs, Beverly, MA, USA) for 14 hrs at 37°C.

**Figure 1:**
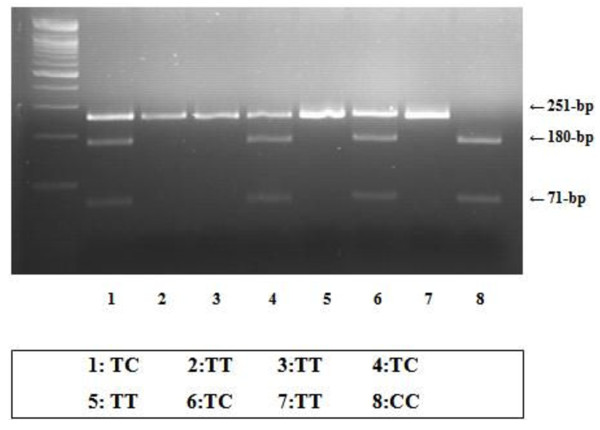
**The -1031(T/C) polymorphism of the *TNF-α *gene**. Shown is 2% agarose gel on the electrophoresis stained with ethidium bromide following *Bbs *I digestion. The 251- and 13-bp indicate the homozygosity of the T allele. However, 13-bp band was not observed in 2% gel. 180-, 71-, and 13-bp indicated the homozygosity of the C allele. And the T/C genotype indicates three bands of 251-, 180-, and 71-bp.

DNA fragments were electrophoresed on 2% agarose gels containing ethidium bromide in 0.5× Tris-Borate-EDTA buffer. Stained fragments were visualized under ultraviolet transilluminator. The fragments of 251- and 13-bp revealed homozygosity for the T allele and 180-, 71- and 13-bp fragments indicated homozygosity for the C allele (Figure 1). In addition, PCR products of each genotype were sequenced to confirm the genotyping results from the RFLP analysis.

### Statistical analysis

Statistical analysis was performed using HapAnalyzer. *X*^2 ^test and logistic regression were utilized to analyze the association between two groups. A *p*-value under 0.05 was considered statistically significant.

## Results

According to the instruction of the ASRM/ESHRE Rotterdam consensus in 2003, PCOS patients showed following two of the three phenotypes: clinical or biochemical hyperandrogenism, oligo- or amenorrhea and polycystic ovarian morphology through ultrasonography. To validate the clinical and biochemical characteristics between PCOS patients and controls, all subjects were measured for body mass index (BMI), waist/hip ratio (WHR), glucose, and hormone levels including FSH, LH, E_2_, PRL, TSH, testosterone and DHEA-S are shown in Table 1. Consequently, the PCOS patient group showed distinct difference compared with the control group. The level of TSH, DHEA-S, and testosterone were slightly higher in the PCOS group. Furthermore, 33 patients (15.2%) in the PCOS group had hyperandrogenism and oligo- or amenorrhea, 18 patients (8.3%) in the PCOS group had hyperandrogenism and polycystic ovaries, 141 patients (65%) in the PCOS group had oligo- or amenorrhea and polycystic ovaries, 22 patients (10.1%) in the PCOS group had hyperandrogenism, oligo- or amenorrhea and polycystic ovaries which are summarized in Table 2.

**Table 2:** Comparison of clinical characteristics between PCOS patients (n = 217) and controls (n = 144)

Characteristics	PCOS patients (n = 217)	Controls (n = 144)
Hyperandrogenism and Oligo- or amenorrhea	n = 33 (15.2%)	n = 0 (0.0%)
Hyperandrogenism and Polycystic ovaries	n = 18 (8.3%)	n = 0 (0.0%)
Oligo- or amenorrhea and polycystic ovaries	n = 141 (65%)	n = 0 (0.0%)
Hyperandrogenism, Oligo- or amenorrhea and polycystic ovaries	n = 22 (10.1%)	n = 0 (0.0%)

**Figure 2:**
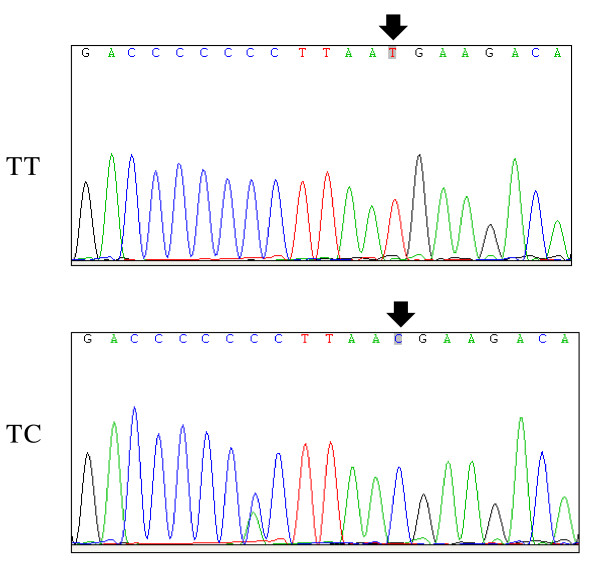
**Sequence analysis for the -1031(T/C) polymorphism of *TNF-α *gene using forward primer**.

For the RFLP analysis for the -1031(T/C) polymorphism of *TNF-α *gene, we recruited 217 patients and 144 control samples. In the present study, the frequency of T/T, T/C, and C/C genotypes of -1031(T/C) polymorphism in TNF-*α *gene was confirmed by sequence analysis and it showed different proportion (*p*-value = 0.0003, odd ratio (OR) = 2.53) between PCOS and control groups (Figure 2, Table 3). According to the X^2 ^test, both groups were in Hardy-Weinberg equilibrium (HWE). The frequency of T/T genotype between PCOS and control groups was 66.3% and 84.7%, respectively. The frequency of T/C genotype between PCOS and control groups was 32.7% and 15.3%, respectively. And, the frequency of C/C genotype between PCOS and control groups was 0.9% and 0%, respectively. Our results demonstrated that there was significant difference between PCOS and control groups, indicating that there is strong association between the -1031(T/C) polymorphism in the promoter region of *TNF-α *gene and PCOS.

**Table 3:** Allele frequencies of -1031(T/C) polymorphism of *TNF-α *gene in PCOS group (n = 217) and control group (n = 144)

	PCOS patient group	Control group
Genotypes, n (%)		

TT	144 (66.5%)	122 (84.7%)

TC	71 (32.7%)	22 (15.3%)

CC	2 (0.9%)	0 (0.0%)
Alleles, n (%)		
T	359 (82.7%)	266 (92.4%)
C	75 (17.3%)	22 (7.6%)
Total	OR (95% CI) = 2.53 (1.53-4.17), *p*-value = 0.0003	

## Discussion

TNF-*α *is a multifunctional proinflammation cytokine and plays an important role in wide-range of various diseases [19,20]. It exists not only in oocytes [21-24] and granulosa cells [25-28], but also in follicular fluid of human ovary [29-31]. It is considered to be related to ovarian apoptosis, increased ovarian steroid secretion, and anovulation [32,33]. Furthermore, it was reported that serum level of TNF- *α *was shown to increase in patients with PCOS. The *TNF-α *gene is known to cause a decrease in insulin receptor tyrosine phosphorylation and an increase in serine phosphorylation of insulin receptor substrate, leading to inhibition of downstream insulin signaling and glucose uptake [34].

TNF-*α *expression is regulated at both the transcriptional and post-transcriptional levels. Moreover, TNF-*α *gene transcription is regulated by the promoter region which consists of an 1100 base pair of DNA [35,36].

Up to now, several studies of the -1031(T/C) polymorphism of *TNF-α *were reported including Behcet's disease [37], large joint arthropathy [38], and Crohn's disease [39]. Crohn's disease and Behcet's disease patients showed the increased frequency of -1031C allele compared with controls. In contrast, patients with ulcerative collitis and hyperandrogenism showed lower frequency of -1031C allele than that of controls [39,40]. In the present study, we investigated the influence of -1031(T/C) polymorphism of *TNF-α *on PCOS by biochemical, clinical, and molecular genetic approaches. Our data showed that the -1031(T/C) polymorphism in the promoter region of *TNF-α *gene is associated with PCOS in a Korean population. Our study indicated that the C allele may provide protection from PCOS, and this promoter polymorphism has been associated with the number of other diseases including inflammatory bowel diseases [41], rheumatoid arthritis [42], and breast cancer [43]. Further studies are required to determine the functional significance of the -1031C allele in PCOS. This is the first study to demonstrate an association between the -1031(T/C) polymorphism in the *TNF-α *gene and PCOS. Genetic predisposition can be investigated with association studies, and our results suggest that it could be one of the etiological factors for PCOS and that the -1031(T/C) polymorphism may play an important role for the progression of PCOS.

Up to now, there is no transcriptional factor binding to the -1031(T/C) polymorphism of *TNF*-*α *gene. It is very important to investigate the molecular mechanism of the polymorphism for PCOS. Further studies are required for the functional significance of the polymorphisms in *TNF-α *and may be suggested to be a credible molecular and immunological marker. And further association studies of PCOS with related genes required for finding the pathogenesis of PCOS and SNPs, which have association with PCOS important for prediction and suggestion as a biomarker for diagnosis of the disease.

In conclusion, this is the first report on the association of -1031(T/C) polymorphism of TNF- *α *gene with PCOS. These data might be very informative for the advancement of future genetic treatment for PCOS patients.

## Competing interests

The authors declare that they have no competing interests.

## Authors' contributions

JY designed the study and drafted the manuscript. JY and JC performed molecular genetic studies. KL and JS designed the study and offered the blood samples. KB designed the study, contributed the data analysis, and wrote the manuscript. All authors read and authorized the final manuscript.
